# Endogenous expression mapping of malignant melanoma by mass spectrometry imaging

**DOI:** 10.1186/s40169-018-0201-x

**Published:** 2018-08-06

**Authors:** Yutaka Sugihara, Daniel Rivas, Johan Malm, Marcell Szasz, HoJeong Kwon, Bo Baldetorp, Håkan Olsson, Christian Ingvar, Melinda Rezeli, Thomas E. Fehniger, György Marko-Varga

**Affiliations:** 1 Clinical Protein Science & Imaging, Biomedical Center, Department of Biomedical Engineering, Lund University, BMC C13, 221 84 Lund, Sweden; 2 Department of Oncology, Clinical Sciences, Lund University, Skåne University Hospital, 221 85 Lund, Sweden; 3 Clinical Protein Science & Imaging, Biomedical Center, Department of Biomedical Engineering, Lund University, BMC D13, 221 84 Lund, Sweden; 4 Section for Clinical Chemistry, Department of Translational Medicine, Lund University, Skåne University Hospital Malmö, 205 02 Malmö, Sweden; 5 Centre of Excellence in Biological and Medical Mass Spectrometry, BioMedical Centre D13, Lund University, 221 85 Lund, Sweden; 6 Cancer Center, Semmelweis University, Budapest, 1091 Hungary; 7 Department of Tumor Biology, National Koranyi Institute of Pulmonology, Budapest, 1121 Hungary; 8 Chemical Genomics Global Research Lab, Department of Biotechnology, College of Life Science and Biotechnology, Yonsei University, Seoul, 120-749 Republic of Korea; 9 Department of Surgery, Clinical Sciences, Lund University, SUS, 221 85 Lund, Sweden

**Keywords:** Malignant melanoma, Cancer, MALDI-MS imaging

## Abstract

**Background:**

Currently, only a limited number of molecular biomarkers for malignant melanoma exist. This is the case for both diagnosing the disease, staging, and efficiently measuring the response to therapy by tracing the progression of disease development and drug impact. There is a great need to identify novel landmarks of disease progression and alterations.

**Methods:**

Matrix-assisted laser desorption ionisation mass spectrometry imaging (MALDI-MSI) has been developed within our group to study drug localisation within micro-environmental tissue compartments. Here, we expand further on this technology development and introduce for the first time melanoma tumour tissues to map metabolite localisation utilising high resolution mass spectrometry. MALDI-MSI can measure and localise the distribution pattern of a number of small molecule metabolites within tissue compartments of tumours isolated from melanoma patients. Data on direct measurements of metabolite identities attained at the local sites in tissue compartments has not been readily available as a measure of a clinical index for most cancer diseases. The current development on the mapping of endogenous molecular expression melanoma tumours by mass spectrometry imaging focuses on the establishment of a cancer tissue preparation process whereby a matrix crystal formation is homogenously built on the tissue surface, providing uniform molecular mapping. We apply this micro-preparation technology to disease presentation by mapping the molecular signatures from patient tumour sections.

**Results:**

We have automated the process with a micro-technological dispensing platform. This provides the basis for thin film generation of the cancer patient tissues prior to imaging screening. Compartmentalisation of the tumour regions are displayed within the image analysis interfaced with histopathological grading and characterisation.

**Conclusions:**

This enables site localisation within the tumour with image mapping to disease target areas such as melanoma cells, macrophages, and lymphocytes.

## Background

Metastatic malignant melanoma is a cancer with a highly unfavourable prognosis and has one of the highest global incidence rates. In Sweden, close to 4000 new patients are diagnosed every year and almost 500 patients die of disseminated melanoma annually. In addition, the 5 year survival rate for metastatic melanoma is around 5% and the median survival is only 6–10 months [1, 2].

In contrast to most other malignancies, malignant melanoma is also common in young people and not only the elderly. Requirements for patient safety and drug efficacy are steadily increasing in modern healthcare and are key factors in modern drug development. Due to the relatively high degree of heterogeneity within the tissue, tumours from melanoma patients are in a category that present a real challenge. Disease presentation alters from tissue to tissue and can range from light in colour and soft in nature to dark brown/black due to pigmentation and very rigid and hard due to the stromal spread amongst the cancer cells. A large amount of lymphatic cells are also present in some of the tumours.

Using MALDI ionisation, mass spectrometry was first demonstrated as a tool capable of providing molecular images of proteins by Caprioli et al. [3–5]. Other types of instruments such as the secondary ion mass spectrometer (SIMS) [6] and the desorption electrospray ionisation (DESI) mass spectrometer [7] can be used for MS imaging (MSI). Nevertheless, today MALDI-MSI is the preferred and most frequently used approach for the localisation of biomolecules and drugs over a wide molecular mass range. The method is not only applicable to protein/peptide localisation; but also, to lipids and low molecular mass compounds [8–10].

The challenge today is to establish a highly reproducible, thin-film, crystal surface on the cancer tissue samples. This is a fundamental requirement to generate a sample amenable to analysis by MALDI-MS imaging. Our research team initially developed manual preparation methods that were based on a thin-film technique that was generated using a multi-layer principle. By establishing an automated, highly-reproducible, thin-layer technology that could be applied to cancer tissues, a mass spectrometry (MALDI) sample target was produced from which high-resolution and high-sensitivity quantitation of drug compounds localised in patient tumours could be obtained [11]. The present study reveals experimental details on tumour tissue preparation technologies that provide optimal imaging properties of small molecule markers [12]. In addition, we demonstrate the expression of multiple melanoma cancer metabolites localisation by MSI [13].

## Methods

### Materials

Water, acetonitrile, methanol (Chromasolv^®^ Plus for HPLC), trifluoroacetic acid (TFA) and α-cyano-4-hydroxycinnamic acid (CHCA) were purchased from Sigma-Aldrich (Steinheim, Germany). Chemicals for histology purposes were purchased from Histolab (Gothenburg, Sweden).

### Clinical material

Human tumour tissue samples were obtained from our local biobank in Lund, Sweden, which includes electronically surveilled samples from the university hospital. Lymph nodes containing metastases of malignant melanoma (stage III) were surgically obtained from melanoma patients undergoing treatment at the Skåne University Hospital, Sweden. The fresh specimens were snap frozen and stored at − 80 °C in the local malignant melanoma biobank until use with the informed consent of each donor. The frozen specimens were used as described below for both mass spectrometry imaging analysis and histological comparison of the same specimens. All cases and samples were reviewed by a pathologist and evaluated for tissue content regarding cancer, immune infiltration and other components.

### Tissue sectioning and sample preparation

10-μm frozen sections were cut using a cryotome and placed onto glass slides. After drying of the tissue, a CHCA (alpha-cyano-4-hydroxycinnamic acid) matrix solution was applied onto the tissue surface. Matrix deposition was done using a solution of 5 mg/mL CHCA in 50% methanol with 0.05% TFA that was sprayed onto the tissue sections with the help of an automated pneumatic sprayer (TM-Sprayer, HTX Technologies). The nozzle distance was 46 mm, and the spraying temperature was set to 75 °C, the matrix was sprayed (18 passes) over the tissue sections at a linear velocity of 750 mm/min with a flow rate set to 0.1 mL/min and a nitrogen pressure set at 10 psi. After each pass, a drying time of 8 s was set on the spraying machine to give time for the sample to dry completely before the next pass.

Histochemistry. Human malignant melanoma tumours were sliced into 10 µm thick sections on a cryotome, dried for 15 min at 37 °C and fixed with 100% MeOH. Tissue sections were stained with conventional Mayers haematoxylin and eosin staining. Digital images of stained sections were acquired with a digital microscope slide scanner (Mirax MIDI, Zeiss, Germany).

### Mass spectrometry imaging of cancer metabolites

MS analysis was performed on a MALDI LTQ Orbitrap XL mass spectrometer (ThermoScientific, Bremen, Germany). Full mass spectra were obtained by using the FT analyser (Orbitrap) at 60,000 resolution (at *m/z* 400). Tissue sections were sampled in positive mode with a 100–1000 Da mass range and 50 μm raster size. The nitrogen laser was operated at 10.0 μJ with automatic gain control (AGC) off mode using 10 laser shots per position. Evaluation of the spectra was performed with Xcalibur v 2.0.7. software, while the visualization of endogenous signals was implemented with the ImageQuest™ software (both from Thermo Fisher Scientific, San José, CA).

## Results

### MALDI matrix application protocol

MALDI-MSI enables the direct analysis of tissue sections. A major advantage of this highly-emergent technology is that the distribution of a large number of analytes can be simultaneously measured without destroying the sample. The MALDI-MSI technology platform provides an accurate identification of endogenous metabolites, lipids, acetylcholine derivatives, peptides and administered compounds (e.g., drugs and small molecules) at exact sampling positions within a tissue section. Prior to the advent of MALDI-MSI, visualising in vivo drug distributions was only possible by incorporating endogenous or exogenous chemical labels into the compounds for subsequent technologies such as autoradiography or positron emission tomography (PET). To monitor the appearance and localisation of these tumour expression signatures, a novel microtechnology tissue imaging preparation platform was used. From this, dedicated tissue preparation methodologies and protocols were developed.

To be able to register the location of the drug ion and/or endogenous signals within the samples, it is important to control that the matrix crystals form a thin film. In the past, we developed thin-film technologies that were implemented into the drug development process in the pharmaceutical industry [14–17].

**Fig. 1:**
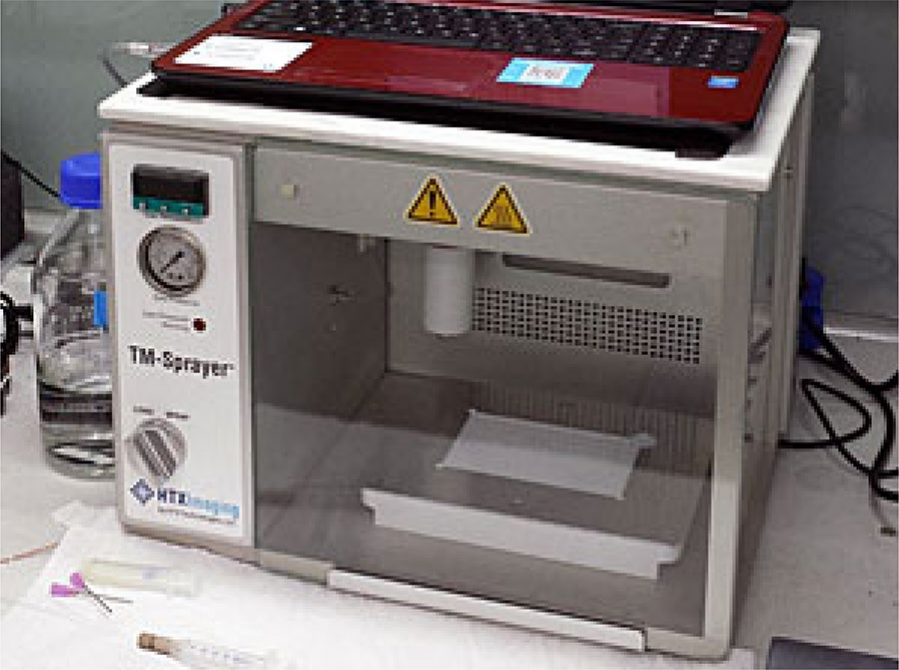
The automated MALDI sample preparation platform, TM-Sprayer

The established work scheme was adapted to cancer tissues. Matrix deposition onto the cancer tissue was performed using an automated MALDI sample preparation platform called the TM-Sprayer (Fig. 1). This system was developed and designed to provide the highest-quality matrix deposition onto two-dimensional biological samples. This MALDI imaging sample preparation workflow was optimised for melanoma tissues and the optimised parameters are represented in Table 1.

**Table 1:** Tissue preparation parameters optimised for melanoma tissues surgically isolated from lymph nodes

Matrix	Matrix (mg/mL)	T (°C)	Passes	Solvent	Flow rate (mL/min)	Velocity (mm/min)	Track space (mm)	Pattern	Drying time (s)
CHCA	5	75	18	50% MeOH/H_2_O 0.05% TFA	0.1	750	3	HH	8

The micro-crystalline matrix platform is shown in Fig. 1, with the tissue station board located within the humidity chamber compartment.

**Fig. 2:**
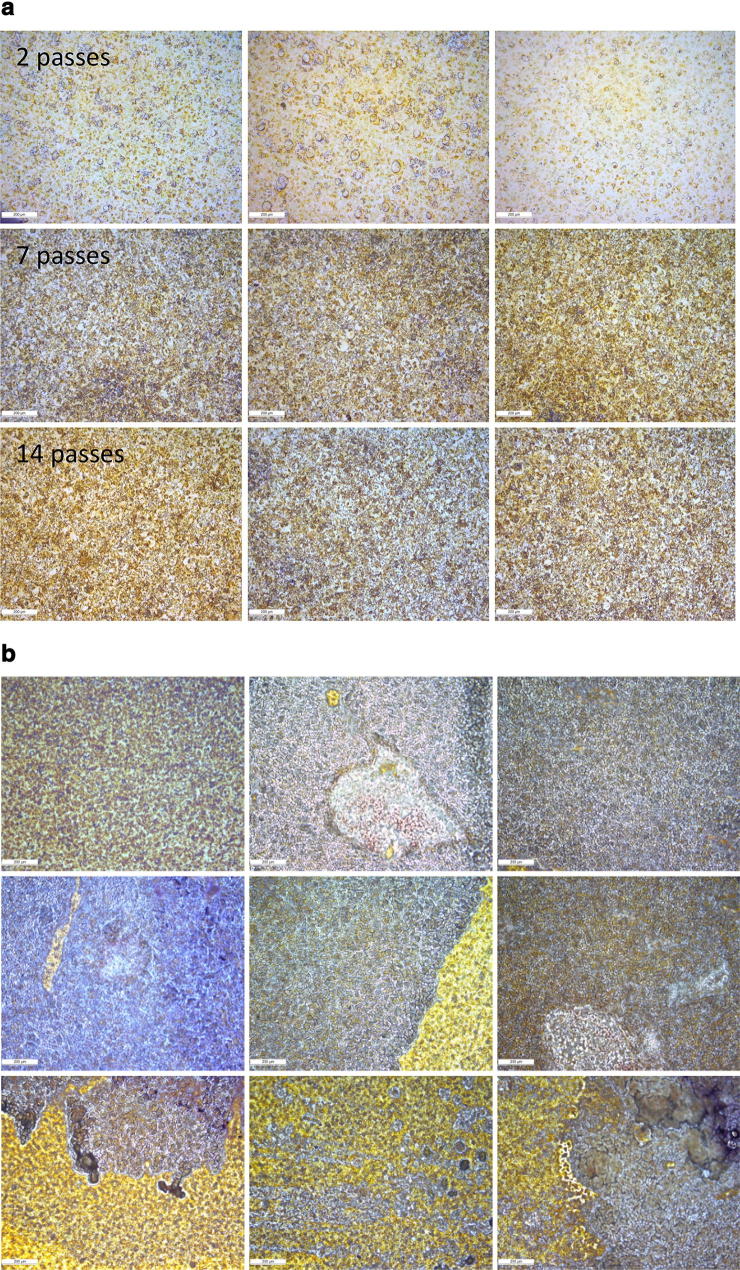
**a** Examples of thin-layer matrix surfaces generated on the glass microscope slide with varying passes of spraying and no tissue (i.e. the number of times the sprayer covers the glass slide area). **b** Examples of thin-layer matrix surfaces generated on melanoma tissues with varying passes of spraying

The MALDI-MS imaging principle is based on the introduction of a UV-activated matrix molecule in the immediate proximity of the analyte. As a consequence, it is of major importance to have complete control of the process by which matrix is introduced onto the sample. As shown in Fig. 1, the TM-Sprayer unit allows heated matrix to be adapted onto the cancer tissue surface. This will lead to an accelerated adsorption onto the tissue and the controlled flow of dry nitrogen to focus the spray, and control of the drying time. The optimal conditions that were developed for melanoma tissues surgically-isolated from lymph nodes is presented in Table 1. By applying the outlined protocol, the resultant MALDI matrix crystal surfaces that were generated are shown in Fig. 2. The increasing number of passes with the instrument is image captured and shown in multiple experiments (see Fig. 2).

The micro-crystals were optimised by running a variety of measurements on the slide section and a high correlation and reproducibility was observed. Examination of the MALDI matrix surfaces revealed that the crystals were highly uniform. To generate high-sensitivity images, this is an important factor. Repeated measurements on the diameter of the crystals identified from the microscope images revealed that the relative statistics of the experiment returned P-values ranging from 0.07 to 0.80 (*t* test calculations). No significant differences were apparent between the blue and yellow crystals; neither across the entire glass slide (Fig. 2a) nor between the spots.

Matrix crystal formation with different matrices was optimised using various cancer tissue samples and ‘protocols’. Samples were analysed by MALDI imaging (MSI) and the intensities of the signals compared to optimise the process (Fig. 2b). The results obtained after optimisation of the sample preparation revealed that the tissue properties have a major impact on the homogeneity of the crystals that form on the surface of the tumour tissue.

### MALDI-MS imaging analysis

**Fig. 3:**
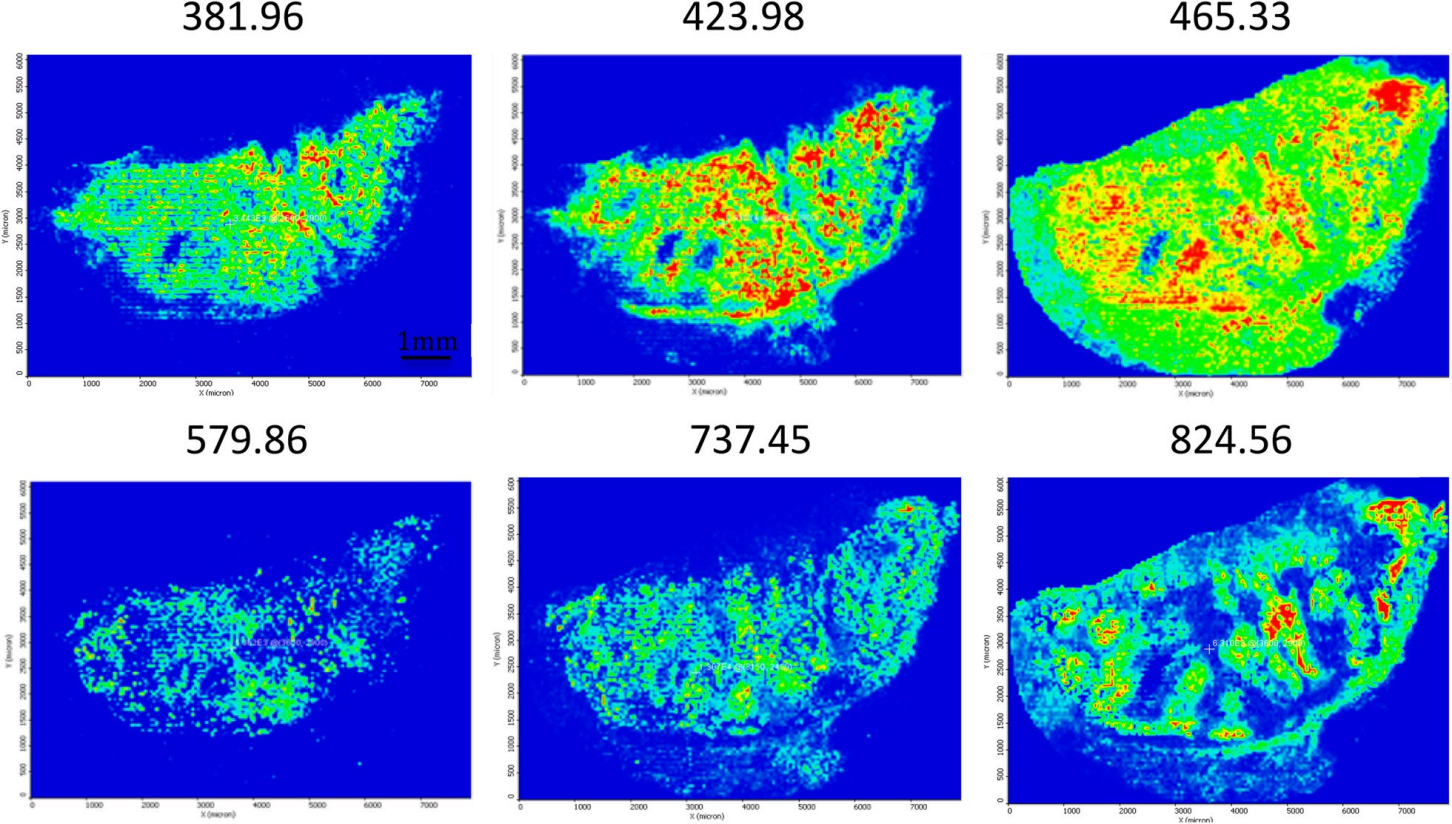
MALDI images from melanoma tissue samples isolated after surgery within a m/z range 100–1000. The scale bar represents 1 mm

By applying our optimised CHCA matrix protocol, the landscape features of the melanoma tissue could be determined. These ranged in mass-to-charge (*m/z*) from 100 to 1000, and are presented in Fig. 3 as a series of image data captures. Shown are representative masses of metabolites detected in the cancer tissue sample together with their tissue distribution (Fig. 3).

For each metabolite, the allocation across the tumor section was shown to vary substantially. Coverage across the entire tissue was apparent for metabolites with both low and high-mass *m/z* values. Among others, ions with m/z values of 381.96, 423.98 and 465.33 appeared to be distributed across the entire tumour region of the tissue. The difference in expression levels, however were obvious and can be seen by observing the yellow/red colours that correspond to high signal responses. Conversely, metabolites at *m/z* 737.45 and 824.56 were only present in specific regions of the tumour. For other ions, however, signal intensity was very low and localised to smaller areas of the tissue, e.g., *m/z* 579.86.

We have established the first, specific landscape reference images from micro-crystalline thin-layer MALDI readouts of endogenous compounds that were observed in metastatic lymph node tissue isolated from melanoma patients and recruited from the South Swedish healthcare oncology biobank. Individual high resolution accurate mass spectra were collected from all measurement points of each analysed tumor tissue section. In all likelihood, these compounds have metabolite properties.

### Compartment image analysis

To register the location of an endogenous signal from a tissue sample, the histological aspect of the patient sample was also implemented. Following MALDI MSI analysis the matrix was removed from the samples and the tissue sections were stained with H&E (haematoxylin & eosin). Digital images of the stained sections were acquired by conventional light microscopy or using a digital microscope slide scanner.

Combined with histological characterisation, several cancer tissues were analysed by MALDI imaging. The pathological analysis identified three major disease-driven regions within specific compartments of the tissues. These were divided into: (i) melanoma cells; (ii) lymphocytes; and (iii) macrophages.

How these have been annotated in our cancer data sets is directly related to the histology section.

**Fig. 4:**
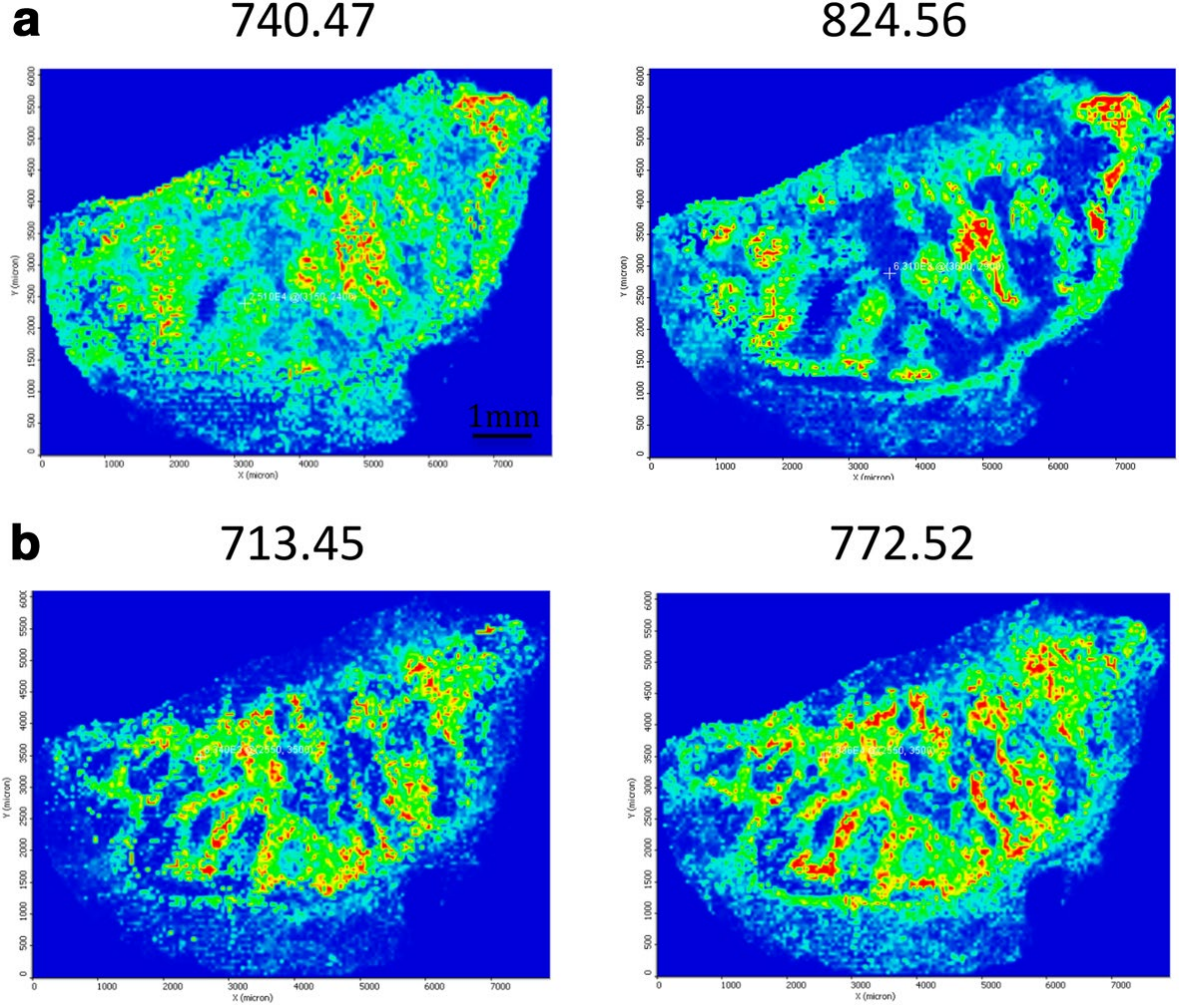
MALDI images from melanoma tissue samples isolated after surgery. Metabolites were detected in melanoma and lymphocyte compartments. The scale bar represents 1 mm

The respective cell types were identified within the tissue section and the areas of the different tissue compartments were manually outlined to create mass lists of the corresponding metabolites for each cell type. These lists were then further investigated to select the most common metabolite signals across the entire tissue section and to find more specific metabolites that are characteristic to a given cell type. We could find several ion peaks whose spatial distribution correlates well with the tissue distribution of the different cell types. Some representative MALDI MS images of different metabolite signals are illustrated on Fig. 4. The precise identification of these characteristic masses was not attempted; rudimentary identification of the peaks was carried out based on accurate mass delivered by a high-resolution Orbitrap MS instrument using database search.

**Fig. 5:**
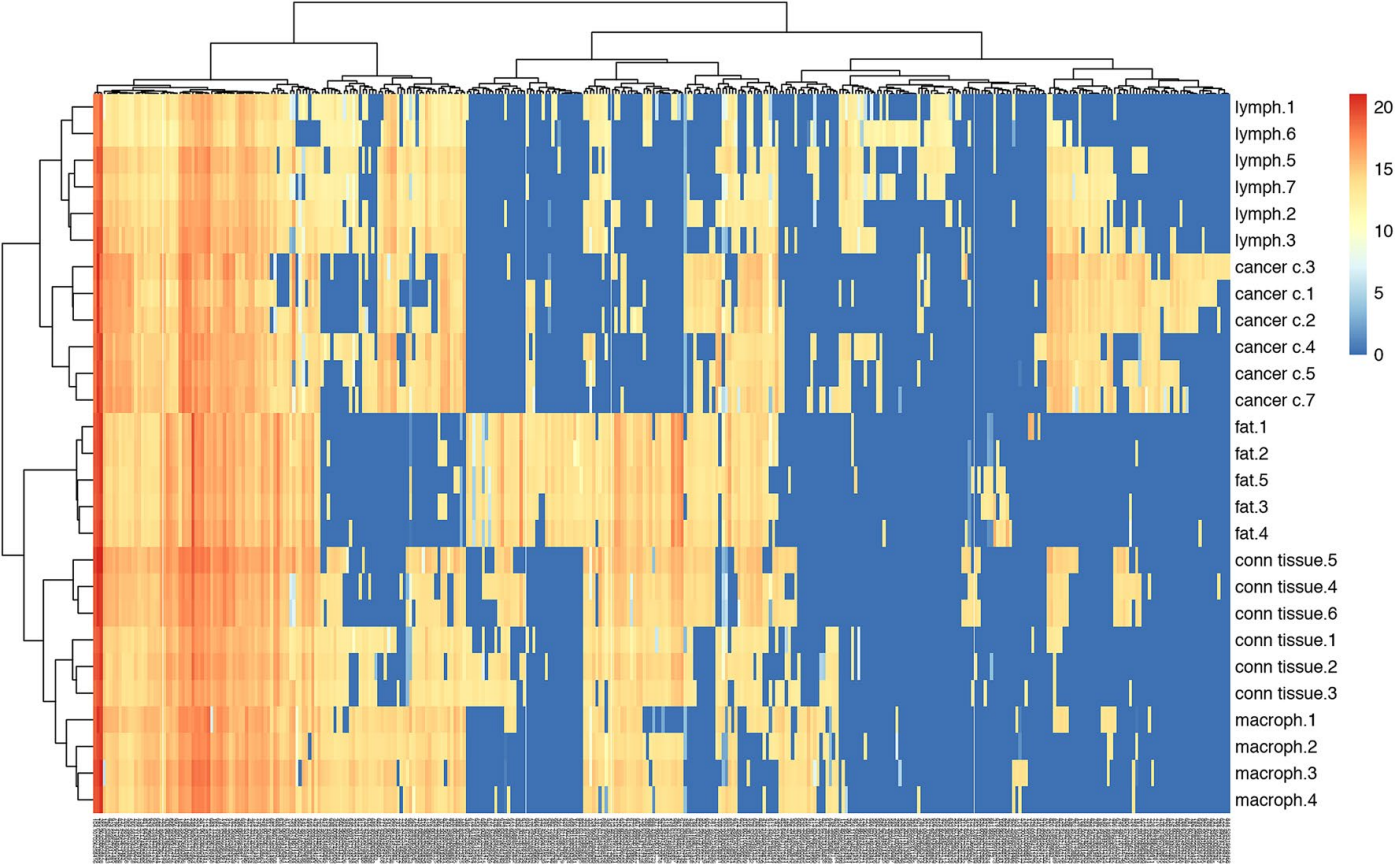
Heat map generated from the metabolite peak lists of the respective cell types in melanoma tissue samples isolated after surgery

After performing a cluster analysis on the peak lists of respective compartments in the tumour tissue, it was obvious that there is a signal pattern that can be specifically correlated to each cell type within the tissue. The heat map generated from all the data is given in Fig. 5.

**Fig. 6:**
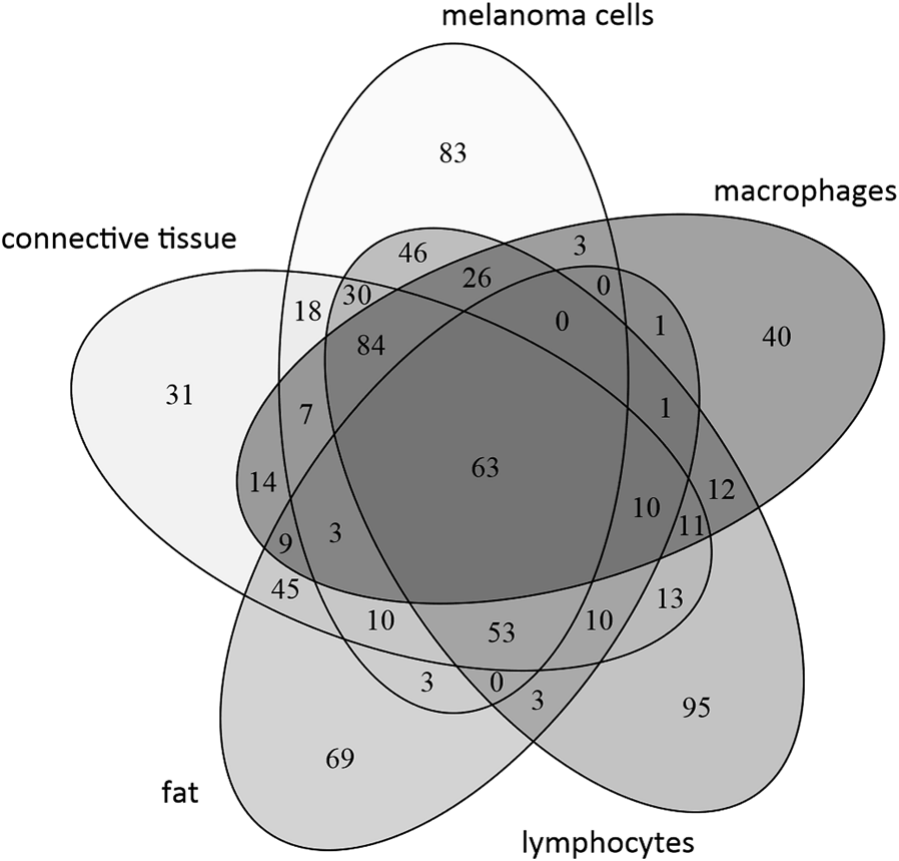
Venn diagram showing the distribution of the detected endogenous signals present in the different regions of the MALDI images from melanoma tissue samples isolated after surgery

From this analysis using MALDIViz (https://omictools.com/maldiviz-2-tool) application it was clear that the overlap of expression is rather high, however, each and every region also has a specific molecular signature that can be correlated to that region of the tissue. The melanoma cells and lymphocytes appear to be the cell types that have the highest number of overlapping molecular ions (Fig. 6).

### Image overlay—coordinating histology with expression

A detailed histopathological characterisation of the patient tissues that includes the morphology and details of the disease presentation in the tumour can be used as a verification for later analysis. Such analysis was performed by matching the histopathological features of the tissue sections with the mass spectrometry images, as the same tissue section was subjected to histological characterization after H&E staining.

**Fig. 7:**
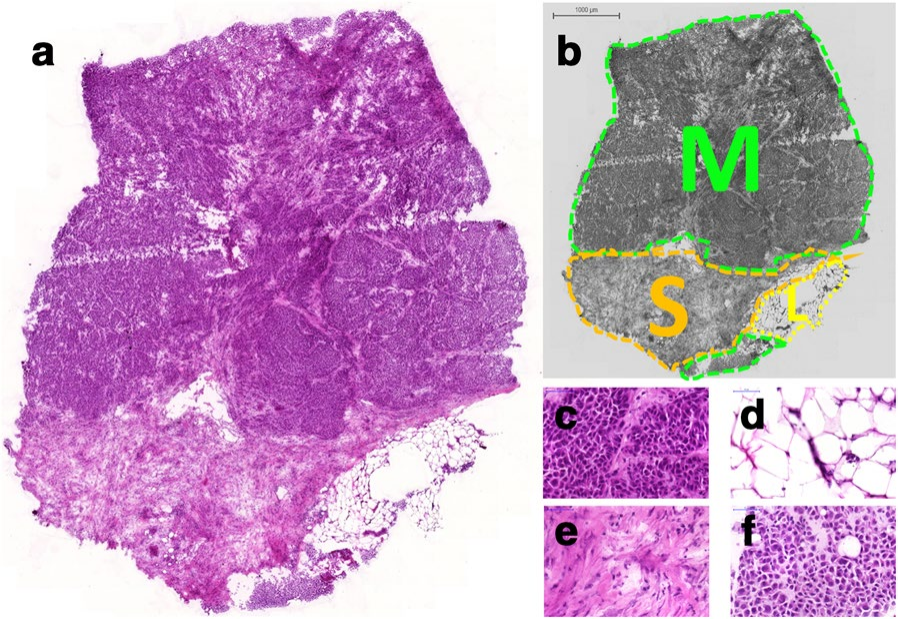
Histological images of a tissue sample displaying: **a** overview with low magnification (1×, HE stain), **b** an annotated area (M: tumour, S: connective tissue, L: fat tissue), **c**–**f** high magnification images of the resultant regions representing (**c**) melanoma from upper region, **d** fat tissue, **e** connective tissue, **f** melanocytes from lower region (53×, HE stains)

As an example, the H&E stained section on Fig. 7 illustrates a melanoma infiltrating subcutaneous tissue. Dermal involvement of atypical/neoplastic melanocytes with cytologic atypia was apparent. The tumour lesion was divided into different areas by morphological feature of the neoplastic melanocytes. In addition to areas containing large melanocytes having abundant cytoplasm and polygonal nuclei, regions containing small cells with minor cytoplasm and small nuclei composed of dense chromatin can also be found. Also, infiltration area of brown pigment laden macrophages was observed in the tumour and the attached subcutaneous tissue.

**Fig. 8:**
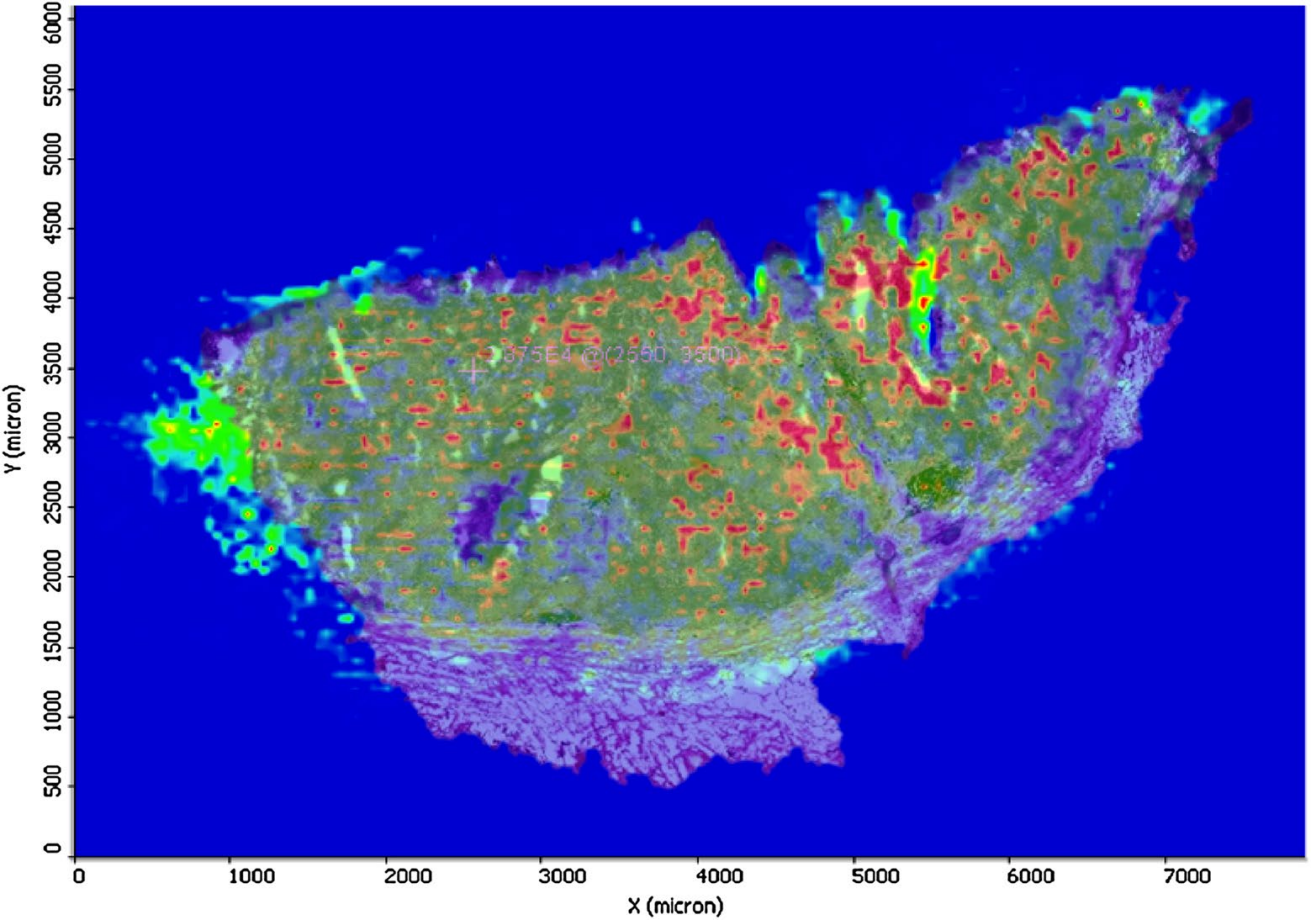
Counterplot of the MALDI image overlaid with the histology image from cancer patient tissue

The images from histology and MALDI imaging can be carefully superimposed. Using landmarks within the images of the tissue sections scanned in MS mode, these were mounted on the histological images of each sample. This is illustrated in a counterplot image as shown in Fig. 8. The borders of known histological compartments in the H&E stained samples could be visually assigned.

MALDI imaging mass spectrometry demonstrated the presence of specific ions in each area. In addition, what is reported here is that the vast majority of the signal defined as metabolite is clearly located within recognisable structures within the tissue.

The present imaging method would be applicable for the biomarker discovery, and also for the characterization of melanoma phenotypes, based upon metabolite expression profiles. Currently we have a software development ongoing that will aid in the determination and diagnosis of melanomas. Ultimately the goal is to improve on personalized treatments. To our knowledge, no other technology except for mass spectrometry imaging is able to characterize the distribution of metabolites with high spatial resolution in a human tissue.

## Concluding remarks

For the first time, we have successfully demonstrated that the current feasibility investigation on direct measurement of metabolites that are located at specific sites in the tumour compartments has potential as a clinical index for malignant melanomas. This is of particular value and importance as precision medicine treatments are rapidly developing as a first line therapy for many cancer types. MALDI-MSI assays that can identify multiple metabolite signals in melanoma tissues will provide much needed information about the distribution and pharmacokinetic properties of pharmaceutical compounds, and the subsequent link to specific disease presentation and development within the clinic.

## Abbreviations

MALDI-MSI: matrix-assisted laser desorption ionisation mass spectrometry imaging
MSI: MS imaging
CHCA: alpha-cyano-4-hydroxycinnamic acid
H&E: hematoxylin & eosin
